# Hypothalamic Expression of Neuropeptide Y (NPY) and Pro-OpioMelanoCortin (POMC) in Adult Male Mice Is Affected by Chronic Exposure to Endocrine Disruptors

**DOI:** 10.3390/metabo11060368

**Published:** 2021-06-09

**Authors:** Marilena Marraudino, Elisabetta Bo, Elisabetta Carlini, Alice Farinetti, Giovanna Ponti, Isabella Zanella, Diego Di Lorenzo, Gian Carlo Panzica, Stefano Gotti

**Affiliations:** 1 Laboratory of Neuroendocrinology, Department of Neuroscience, University of Torino, 10126 Torino, Italy; marilena.marraudino@unito.it (M.M.); bo.elisabetta@gmail.com (E.B.); carlinielisabetta@hotmail.it (E.C.); alice.farinetti@unito.it (A.F.); giancarlo.panzica@unito.it (G.C.P.); 2 Neuroscience Institute Cavalieri-Ottolenghi (NICO), 10043 Orbassano, Italy; gponti2@gmail.com; 3 Department of Molecular and Translational Medicine, University of Brescia, 25121 Brescia, Italy; isabella.zanella@unibs.it; 4 Clinical Chemistry Laboratory, Diagnostic Department, ASST Spedali Civili di Brescia, 25123 Brescia, Italy; diego.dilorenzo@asst-spedalicivili.it

**Keywords:** endocrine disrupting chemicals, bisphenol A, diethylstilbestrol, tributyltin, neuropeptide Y, pro-opiomelanocortin

## Abstract

In the arcuate nucleus, neuropeptide Y (NPY) neurons, increase food intake and decrease energy expenditure, and control the activity of pro-opiomelanocortin (POMC) neurons, that decrease food intake and increase energy expenditure. Both systems project to other hypothalamic nuclei such as the paraventricular and dorsomedial hypothalamic nuclei. Endocrine disrupting chemicals (EDCs) are environmental contaminants that alter the endocrine system causing adverse health effects in an intact organism or its progeny. We investigated the effects of long-term exposure to some EDCs on the hypothalamic NPY and POMC systems of adult male mice that had been previously demonstrated to be a target of some of these EDCs after short-term exposure. Animals were chronically fed for four months with a phytoestrogen-free diet containing two different concentrations of bisphenol A, diethylstilbestrol, tributyltin, or E_2_. At the end, brains were processed for NPY and POMC immunohistochemistry and quantitatively analyzed. In the arcuate and dorsomedial nuclei, both NPY and POMC immunoreactivity showed a statistically significant decrease. In the paraventricular nucleus, only the NPY system was affected, while the POMC system was not affected. Finally, in the VMH the NPY system was affected whereas no POMC immunoreactive material was observed. These results indicate that adult exposure to different EDCs may alter the hypothalamic circuits that control food intake and energy metabolism.

## 1. Introduction

Two neurochemically distinct sets of hypothalamic neurons controlling food intake are located in the arcuate nucleus (ARC). One group expresses neuropeptide Y (NPY) and agouti-related protein (AgRP). The NPY release by these neurons results in increased food intake and decreased energy expenditure. The other group expresses cocaine- and amphetamine-regulated transcript (CART) and pro-opiomelanocortin POMC, which is processed to melanocortin peptides, such as α-melanocyte-stimulating hormone (α-MSH). The activation of these neurons decreases food intake and increases energy expenditure [1] with an opposite effect of the NPY/AgRP system. Interactions between these two populations allow the NPY neurons to control the activity of the POMC cells. NPY/AgRP and POMC/CART neuronal projections reach hypothalamic nuclei such as the paraventricular nucleus (PVN), dorsomedial hypothalamic nucleus (DMH), and perifornical area [2]. These secondary centers process information regarding energy homeostasis.

Many factors can influence the activity of this system (for example the secretion of leptin by adipocytes), but estrogenic signaling may intersect at several levels with the hypothalamic circuits controlling food intake [3]. In fact, estradiol is involved in the regulation of metabolism through the modulation of food intake, body weight, glucose/insulin balance, body fat distribution, lipogenesis, lipolysis, and energy consumption [4]. The estradiol regulates neuroendocrine circuits controlling the metabolism [5] by acting on the POMC neurons through the estrogen receptor α (ERα) and on the NPY cells through an estrogen-activated membrane receptor, Gq-mER [6]. Indeed, estradiol has an inhibitor function on food intake, repressing the synthesis of NPY and AgRP [7]. Moreover, it seems that the leptin (secreted by adipocytes in proportion to fat mass and the activator of anorexigenic signals) has a common pathway with estradiol to regulate energy metabolism, namely the STAT3 pathway in POMC neurons [7]. Peripherally E_2_ increases both leptin mRNA expression in 3T3 adipocytes and leptin secretion in omental adipose tissue [8]. Alternatively, lack of E_2_ after ovariectomy may affect body weight regulation at a central level and mice deficient in ERα show a marked increase of adipose tissue [9]. There is also some evidence that ovariectomy increases hypothalamic NPY expression and decreases CRH immunoreactivity, promoting hyperphagia [10]. Moreover, E_2_ deficiency causes central leptin insensitivity [9].

Endocrine-disrupting chemicals (EDCs) are industrial pollutants or natural molecules, which can be found as contaminants in the environment. They can interact with natural hormones by mimicking, antagonizing, or altering their actions [11] and may interfere with several brain circuits [12]. Recent evidence from many laboratories has shown that a variety of environmental EDCs (now called metabolic disrupting chemicals, MDCs) can influence adipogenesis and obesity and these effects may be partly mediated by sex steroid dysregulation due to the exposure to these substances and by alterations of nervous circuits involved in the control of food intake and energy metabolism [13,14].

In the present study, we analyzed three widely diffused MDCs—bisphenol A (BPA), diethylstilbestrol (DES), and tributyltin (TBT).

The BPA, one of the most diffused chemicals in the world, is a xenoestrogen present in a very large number of products and may affect multiple endocrine pathways, due to its ability to bind classical estrogen receptors (particularly ER-α) and non-classical ones (membrane receptors) [15], as well as the G-protein-coupled receptor 30 (GPR30) [16]. BPA can also act through non-genomic pathways [17] and bind to a variety of other hormone receptors (e.g., androgen receptor, thyroid hormone receptor, glucocorticoid receptor, and PPARγ) [18]. In vitro experiments have demonstrated that BPA may dysregulate NPY, AgRP, and POMC expression in hypothalamic immortalized cell lines [19,20,21].

The DES is a powerful nonsteroidal synthetic estrogen (pharmaceutical) used until the early 70s to prevent miscarriage in pregnant women. Later this compound was recognized as a cause of reproductive cancers, genital malformations, and infertility in sons or daughters that had been exposed to this drug in utero [22], but it is still in use for veterinary purposes in some countries and is bioaccumulated in the environment [23]. DES exerts an agonistic effect against ER-α and an antagonistic effect against estrogen-related receptor-γ (ERR-γ) [24]. In ovariectomized female rats exposed to an isoflavone-rich diet, DES had no effect on hypothalamic NPY mRNA and increased POMC mRNA [25].

TBT belongs to the EDC family of organotins, it has been employed primarily as an antifouling agent in paint for boats. Other uses are as a fungicide on food crops, and an antifungal agent in wood treatments and industrial and textile water systems [26]. Due to its use in paint for boats, TBT has exerted toxicological effects on marine organisms. For example, TBT can induce masculinization in fish species [27]. Humans are exposed to TBT largely through contaminated dietary sources (seafood and shellfish [28]). In mammals TBT can increase body weight [29], alter hypothalamic NPY and POMC systems in short-term (4 weeks) exposed adult mice [30,31], and may also alter behavior—exposure to a low dose of TBT induced lower activity, high level of anxiety, and fear in mice [32]. TBT binds with high affinity to steroid receptors; in particular, it binds androgen receptor [33] and interferes with the expression of brain aromatase and estrogen receptors [34]. TBT can act as an agonist of retinoid X receptor (RXR) and peroxisome proliferator-activated receptor-γ (PPARγ) [35]. This inappropriate receptor activation could lead to disruption of the normal developmental and homeostatic controls over adipogenesis and energy balance, especially under the influence of the typical high-fat Western diet [36]. In addition, changes in the microbiome are associated with TBT exposure [37].

As previously reported, studies on the action of EDC on hypothalamic neurons related to eating behavior and energy control used a variety of experimental conditions (exposure to isoflavones, in vitro experiments, and short-term exposure). For this reason, in the present study, we exposed, for a longer time period (4 months), adult male mice to phytoestrogen-free food containing different putative MDCs to understand if the central neuroendocrine, orexinergic, and anorexinergic circuits are differentially affected by these compounds. Due to the alleged xenoestrogenic activity of some of them we also included, as a positive control, a group of animals treated with E_2_.

## 2. Results

### 2.1. Body Weight

At the end of the experiment the animals were weighted. Data collected showed a global effect of treatment on the body weight of exposed animals (*p* < 0.05, F_(8)_ = 2.185). In particular, the post-hoc analysis with Fisher’s LSD test showed a reduction in body weight for mice treated with the higher dose of DES (*p* < 0.05) and for those treated with both doses of E_2_ (*p* < 0.05). No statistically significant effects were observed in the other groups (see Table 1).

### 2.2. Immunohistochemistry

#### 2.2.1. NPY System

**Figure 1:**
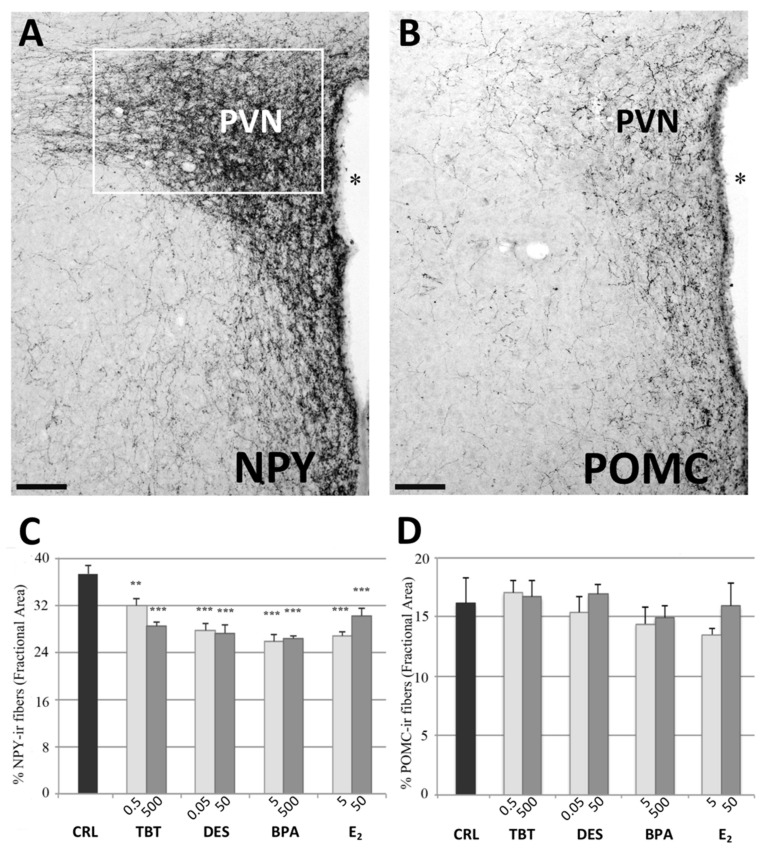
NPY and POMC immunohistochemistry in the PVN. Microphotographs and histograms illustrating the immunohistochemical immunoreactivity for NPY and POMC in the paraventricular nucleus (PVN). (**A**) Low magnification of a control mouse (CRL) illustrating the NPY immunoreactivity in PVN. The white box represent the ROI selected for the quantitative analysis. (**B**) Low magnification of a control mouse (CRL) illustrating the POMC immunoreactivity in PVN. Scale bar = 100 μm. * = Third ventricle. (**C**,**D**) Histograms illustrating the quantitative analysis of the fractional area covered by NPY (C) and POMC (D) immunoreactivity in the PVN. Bars represent the mean and the standard error of the mean (SEM). Asterisks indicate significant differences (Fisher’s test) of the experimental groups in comparison to controls (CRL): ** *p* < 0.01, *** *p* < 0.001.

**Figure 2:**
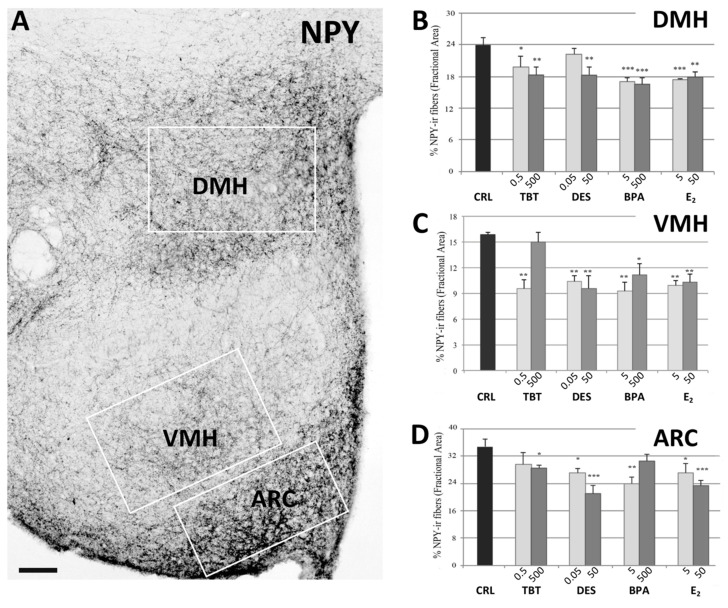
NPY immunohistochemistry. Microphotograph and histograms illustrating the immunohistochemical immunoreactivity for NPY in the dorsomedial (DMH), ventromedial (VMH), and arcuate (ARC) nuclei. (**A**) Low magnification of the hypothalamic region of a control mouse (CRL) illustrating the NPY immunoreactivity in DMH, VMH, and ARC nuclei. The white boxes represent the ROI selected for each nucleus in the quantitative analysis. Scale bar = 100 μm. (**B**–**D**) Histograms illustrating the quantitative analysis of the fractional area covered by NPY immunoreactivity in the DMH (B), VMH (C), and ARC (D) nuclei in the different experimental groups. Bars represent the mean and the standard error of the mean (SEM). Asterisks indicate significant differences (Fisher’s test) of the experimental groups in comparison to controls (CRL): * *p* < 0.05, ** *p* < 0.01, *** *p* < 0.001.

A preliminary qualitative analysis showed a distribution similar to those already reported in previous contributions [30,38,39,40,41]. In particular, we did not observe positive cell bodies (confirming previous reports that NPY cell bodies in ARC are visible only after colchicine treatment [42]), whereas a large number of positive fibers was observed along the entire hypothalamus. These were particularly dense within the PVN (Figure 1) and the ARC (Figure 2) nuclei, but they were also abundant within the suprachiasmatic, supraoptic, and DMH (Figure 2) nuclei. Other regions displayed less dense innervations, as for example, the VMH (Figure 2). In the experimental groups, we observed a qualitative decrease of the NPY immunoreactivity (ir) in all the considered nuclei for all the different treatments.

This qualitative impression was confirmed by the statistical analysis. For all nuclei we found a statistically significant effect of treatment (PVN: *p* < 0.001, F_(8)_ = 10.672; ARC: *p* < 0.01, F_(8)_ = 3.566; DMH: *p* < 0.01, F_(8)_ = 3.767; VMH: *p* < 0.001, F_(8)_ = 5.780).

The post-hoc analysis with Fisher’s LSD test showed a significant decrease of NPYir in all nuclei and for almost all the treatments. In PVN, all groups showed a significantly lower NPYir than controls (*p* < 0.01, Figure 1). In ARC, we did not observe statistically significant differences for the lowest dose of TBT and the highest dose of BPA, while all the other treatments induced a significant decrease of NPY expression (*p* < 0.05, Figure 2). In DMH we observed a significant reduction of NPYir in all treated groups (*p* < 0.05; Figure 2), except for the lowest dose of DES. Finally, in VMH we observed a strong reduction of NPYir due to the treatments (*p* < 0.01) except for the highest dose of TBT (for details see Table S2, Supplementary Materials).

#### 2.2.2. POMC System

**Figure 3:**
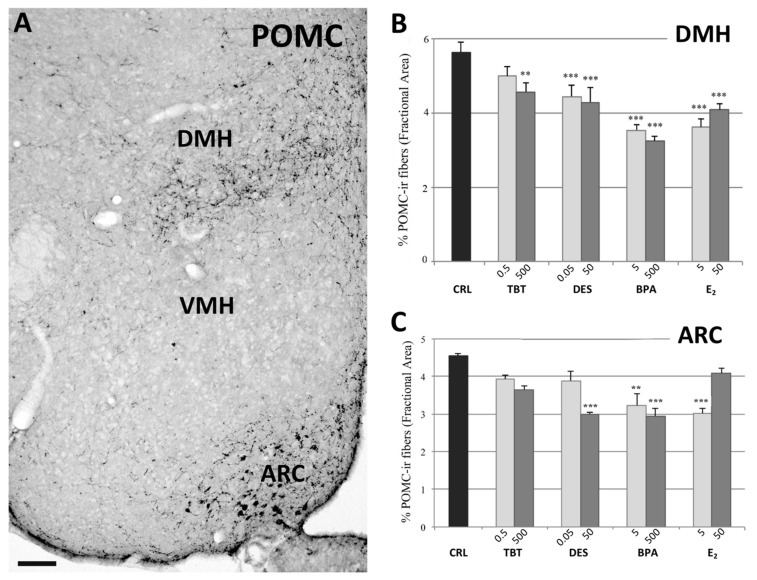
POMC immunohistochemistry. Microphotograph and histograms illustrating the immunohistochemical immunoreactivity for POMC in the dorsomedial (DMH), and arcuate (ARC) nuclei. (**A**) Low magnification of the hypothalamic region of a control mouse (CRL) illustrating the POMC immunoreactivity in DMH, and ARC nuclei. Due to the extreme paucity of immunoreactive structures, it was not possible to measure POMC immunoreactivity in the VMH. Scale bar = 100 μm. (**B**,**C**) Histograms illustrating the quantitative analysis of the fractional area covered by POMC immunoreactivity in the DMH (B), and ARC (C) nuclei in the different experimental groups. Bars represent the mean and the standard error of the mean (SEM). Asterisks indicate significant differences (Fisher’s test) of the experimental groups in comparison to controls (CRL): ** *p* < 0.01, *** *p* < 0.001.

The distribution of POMCir in control mice was in agreement with the few previous studies that described this system in rats [43,44,45] and mice [31,46]. Contrary to NPY, hypothalamic POMC cell bodies are clearly visible, and they were fully included within the rostrocaudal extent of the ARC (Figure 3) and periarcuate area, which also showed a local dense innervation of ir fibers. Two major targets of this system are the PVN and the DMH. In the PVN, POMCir fibers outlined the entire nucleus, starting from its rostral portion up to the more caudal levels. The distribution of these fibers was not homogeneous, in particular they were denser in the medial PVN (corresponding to the parvocellular regions of this nucleus) compared to the lateral PVN (corresponding to the magnocellular region) (Figure 1). The DMH nucleus (Figure 3) showed a denser innervation in the caudal part of the nucleus compared with the rostral part. Other hypothalamic nuclei, such as the VMH, did not show a significant number of positive fibers.

In the PVN we did not observe variations due to treatment. In fact, the statistical analysis showed no effect of treatment (F =1.097, *p* = 0.396, Figure 1). On the contrary, data collected in the ARC showed a decrease of the POMCir (including positive cell bodies and fibers), following the different treatments (F_(8)_ = 8.289, *p* < 0.001). The Fisher LSD test showed a statistically significant decrease in the groups treated with the highest dose of DES (*p* < 0.001), the lowest of E_2_ (*p* < 0.001) and in both groups treated with BPA (*p* < 0.001, Figure 3).

The quantitative analysis also showed a decrease of the POMCir in the DMH following different treatments (F_(8)_ = 19.563, *p* < 0.001). The Fisher LSD test showed a decrease of POMC ir in all groups compared to controls, except for the lowest dose of TBT (Figure 3; for details see Table S2, Supplementary Materials).

## 3. Discussion

The control of energy metabolism and food intake is in part dependent on central neuroendocrine circuits that have been detailed in the introduction. Among the various systems, the NPY and the POMC systems (both located in the hypothalamic arcuate nucleus and sending their axons to other hypothalamic nuclei) exert orexigenic (NPY) and anorexigenic (POMC) effects. Several studies (recently reviewed by [14]) demonstrated that these neural circuits are altered when the animals are exposed to some environmental compounds that are now classified as metabolism-disrupting chemicals (MDCs) [13,47].

In the present study, we showed that some of the putative MDCs, when chronically administered through a phytoestrogen-free diet (reported in the literature as inducing body weight gain [48]), affected the expression of both NPY and POMC in the hypothalamic circuits of adult male mice. For comparison, we included two additional groups, one without any treatment (control group) and the second one exposed to E_2_ (added to the diet), which has a well-known anti-adipogenic effect [49,50].

As expected, in the present experiment, both doses of E_2_ induced a significant reduction of the body weight in comparison to the control group. On the contrary, male mice fed with the same diet but with different concentrations of three different EDCs, except the group treated with the highest dose of DES, did not show any significant reduction of the body weight. These results suggest that BPA, DES, and TBT are not able, in adult male mice, to counteract the consequence of an exposure to a phytoestrogen-free diet on the body weight, whereas E_2_ is able to do this. Therefore, whereas E_2_ has an anti-obesogenic effect, the EDCs considered in this study do not show this property. It is possible that the lack of effect on body weight is due to the fact that the reduction of the activity of the orexinergic circuits originated by the reduction of NPY is compensated by the reduction of the activity of the anorexinergic circuits caused by the reduction of the expression of POMC.

Our data show that the NPY expression in male mice hypothalamic nuclei involved in food intake regulation is reduced by E_2_ as well as by all tested EDCs at almost all doses. Therefore DES, BPA, and TBT have the same effect of E_2_ on the NPY system. In particular, DES and BPA have a well-known strong xenoestrogenic activity because they specifically bind to ERs [51]. On the contrary, TBT does not bind ERs, but it also has xenoandrogenic or antiandrogenic activity [52]. The reduction of NPY expression in the hypothalamus after TBT treatment confirms our previous results [30] and may be due to the activation of other pathways, not directly regulated by E_2_.

The effects of treatments on the POMC system of male mice are less homogeneous. In fact, we observed significant effects on ARC and DMH, while in the PVN we have not detected significant effects. DES, BPA, and also E_2_ significantly decreased the POMC expression in ARC, while TBT showed no significant effect. It is important to note that 30% of POMC cells in ARC colocalize with ERα while they do not express ERβ [53], thus suggesting a possible direct role of ERs in regulating part of this system that represents, consequently, a putative target for xenoestrogens, like BPA and DES. The lack of TBT effect is also in line with our recent results that showed no effects of TBT on the POMC system in adult male mice [31].

The POMC neurons of the ARC send axons to two main targets, the DMH and the PVN. All treatments (including TBT at the highest dose) induced a significant decrease in the immunoreactivity in the DMH, whereas no effect was detected in the PVN, even when the quantitative analysis was performed on the different parts of the PVN, according to the method detailed in [54] (results summarized in Figure S3 of Supplementary Material). It is still possible that the paucity of POMC fibers in the PVN (compared to the NPY ones) has prevented the detection of small differences in the present experimental material.

In a limited number of experimental groups, the tested EDCs showed a significant effect in reducing immunoreactivity at the low dose and not at the high dose, for example see the effect of TBT on VMH NPY immunoreactivity, the effect of BPA on ARC NPY immunoreactivity, or the effect of E_2_ on ARC POMC immunoreactivity. These results confirm the nonmonotonic dose response described in many experimental situations for several EDCs [55]. The differences of the results of EDC treatments on NPY and POMC immunoreactivity with those obtained with E_2_, are probably due to the activation of pathways not directly or indirectly regulated by E_2_. For example, it has been found that intracerebroventricular injections of oxytocin (OT) in adult fasted male rats decreases food intake [56]. Moreover, a retrograde tracer study revealed OT projection from PVN and SON to ARC, demonstrating that oxytocinergic signaling may regulate feeding [57]. OT cells, expressing ER-β, of the PVN [58], are a possible target for xenoestrogen that binds ER-β, like the phytoestrogen genistein [59]. This suggests that some EDCs may alter POMC expression via the OT system. However, the physiological significance of the OT neuronal projections from PVN and SON to ARC POMC neurons, still remains unclear, and further studies are required to clarify it.

One of the most important regulators of the NPY [60] and POMC [61] systems is represented by the cannabinoid receptor CB1. Some EDCs may modulate the expression of this receptor: prolonged exposure to DES produced a reduction in the mRNA for CB1 receptor in the rat pituitary [62], while BPA caused a downregulation of CB1 receptor in the mice hypothalamus [63]. No data are yet available for an action of TBT on the expression of CB1 receptor. Therefore, it is possible that present results on the alterations of NPY and POMC systems are partly due to an effect of the EDCs on the expression of CB1 receptor and a consequent functional alteration of these two systems. Future work should clarify this aspect.

The levels of circulating glucose are also important in controlling the NPY and POMC circuits, through glucose sensitive neurons located in the VMH and LH (for a recent review see [64]). All the three EDCs analyzed in this study disrupt glucose homeostasis by acting on pancreatic islets [37,65,66]. Even if in the present study we have not detected glucose blood levels, it is therefore possible that part of the dysregulation of the NPY and POMC systems is due to alterations of glucose homeostasis.

Another crucial point is that we do not know, at the moment, if we are observing an activational or an organizational effect of these EDCs. In the first case we may expect that the differences in the expression of immunoreactivity are due to an increase or a decrease in the production of neuropeptides in stable circuits (see the effects of BPA on NPY mRNA in neuronal cell cultures [20]). In the second case the hypothesis is that the exposure to the EDCs may induce permanent (or long-term) changes in the observed circuits. In fact, it has been demonstrated that gonadal hormones produced during puberty are inducing neurogenesis in some hypothalamic [67] or extrahypothalamic [68] nuclei and that this process is necessary to stabilize the sexual differences evidenced in these nuclei. A recent review [69] analyzed the available data for the development of hypothalamic circuits that control food intake and energy balance. In summary, in these circuits neurogenesis is only present during the prenatal period [70], but the full maturation of the connections ARC–PVN is reached during the postnatal days 28–35 [71]. However, more recent studies demonstrated that adult neurogenesis of NPY and POMC neurons in mice ARC is stimulated by changes among high fat–low fat diets [72]. Being our animal was three weeks old, it is therefore possible that exposure to EDCs had altered the connection of ARC towards VMH, DMH, and PVN, or even determined a change in the number of NPY and POMC neurons (BPA may induce apoptosis in hippocampal cells [73]). According to this hypothesis the observed changes in the immunoreactivity could be linked to an alteration (plasticity) of fibers’ system reaching these nuclei. At the moment it is impossible to know if NPY and POMC circuits, after such a long exposure to EDCs, when provided with EDCs-free food, may recover to a status comparable to the non-treated animals (this is compatible with an activational effect). Future studies should elucidate this point, in particular not only if there is a recovery, but also how long it will take to recover.

In conclusion, these data, together with those already present in the literature, suggest that EDCs may alter energy metabolism not only at the level of peripheral tissues [13], but also in neuroendocrine circuits involved in the control of food intake, in particular, the NPY and POMC systems. The control of physiological processes by these systems is highly complex, making the understanding of neuroendocrine disruption a particular challenge.

## 4. Materials and Methods

### 4.1. Animals and Treatment

The procedures involving animals and their care were performed in Brescia according to the Union Council Directive of 22 September 2010 (2010/63/UE). The study was approved by the Ethical Committee of Animal Experimentation of the Hospital and the Italian Minister of Health (407/2018-PR). All care was taken to use the minimum number of animals.

C57BJ/6 male mice (Harlan, Udine) were housed in same-sex groups of 4 per cage on a 12:12-h light/dark cycle; animal rooms were maintained at a temperature of 23 °C. Estrogen-free diet was purchased from Dottori Piccioni S.r.L. Via Guglielmo Marconi, 29/31 Gessate (MI, Italy) (https://totofood.it/, assessed on 7 June 2021). The diet was prepared in pellets (the composition is reported in Table S1, Supplementary Materials).

The treatment started when mice were three weeks old and lasted for four months. Animals were divided randomly in nine experimental groups: control mice were fed with the base diet (estrogen-free diet) while experimental groups were fed with the base diet enriched with two different concentrations of E_2_, BPA, DES, or TBT (according to previous studies [74]). All the chemicals were obtained from Sigma-Aldrich, Milano, Italy, dissolved in DMSO and further diluted before their addition to the diet, for homogeneous preparations. These are the doses used: E_2_ (stock solution 97%, cat. number E8515; 5 or 50 µg/kg diet); BPA (stock solution 99%, cat. number 239658; 5 or 500 µg/kg diet); DES (stock solution 99%, cat. number D-4628; 0.05 or 50 µg/kg diet); and TBT (stock solution 96%, cat. number T50202; 0.5 or 500 µg/kg diet).

The normal food consumption in adult mice corresponds to 15g/100g body weight/day [75]; since mice used in this experiment had a mean body weight of 30g, it was considered an approximate consumption of 4.5 g food/day was appropriate. Accordingly, in this case mice were exposed daily to approximately 0.15–1.5 µg/g body weight of E_2_, 0.15–15 µg/g body weight of BPA, 0.0015–1.5 µg/g body weight of DES, and 0.015–15 µg/g body weight of TBT.

Body weights were recorded at the end of the experiment, before sacrifice (see Table 1).

Food consumption was monitored every two days as the difference between the weight of the pellets supplied and that consumed. Spilled food, if any, was collected in apposite trays underneath the food containers, measured, and taken into account.

### 4.2. Tissue Sampling and Histological Examination

Four months after the beginning of treatment adult mice were deeply anesthetized with an intraperitoneal injection of a mixture of ketamine (100 mg/kg of body weight, Ketavet, Gelling, Italy) and xylazine (10 mg/kg of body weight, Rompun, Bayer, Germany) solution, monitored until the pedal reflex was abolished and killed by cervical dislocation. Animals were decapitated, brains were quickly dissected and placed into acrolein (5% in 0.01 M saline phosphate buffer, PBS) for 150 min at room temperature. Brains were rinsed several times in PBS, placed overnight in a 30% sucrose solution in PBS at 4 °C, frozen in liquid isopentane at −40 °C and stored in a deep freezer at −80 °C until sectioning.

Brains (N = 4 for each group) were serially cut in the coronal plane with a cryostat (Leica CM 1900) at 25 µm of thickness. Sections were collected in four series for free-floating procedure in multiwell dishes, filled with a cryoprotectant solution [76] and stored at −20 °C until used for immunohistochemistry. One series of sections was stained for NPY immunohistochemistry and another for POMC immunohistochemistry. Brain sections were always stained in groups containing each treatment, so that between-assay variance could not cause systematic group differences.

After overnight washing in PBS, sections were incubated in 0.01% sodium borohydride for 20 min to remove the acrolein and rinsed in PBS several times. Then, sections were exposed to Triton X-100 (0.2% in PBS) for 30 min and treated for blocking endogenous peroxidase activity with PBS solution containing methanol/hydrogen peroxide for 20 min. Sections were afterwards incubated with normal goat serum (Vector Laboratories, Burlingame, CA, USA) for 30 min. One series was incubated overnight at 4 °C with the rabbit polyclonal antibody against synthetic porcine NPY (gift by Professor Vaudry, France) diluted 1:5000 in 0.2% PBS-Triton X-100, pH 7.3–7.4 and another with the rabbit polyclonal antibody against POMC (Phoenix Pharmaceuticals, Inc.,Burlingame, CA USA) [31,77,78] diluted 1:5000 in 0.2% PBS-Triton X-100 and 1% of BSA, pH 7.3–7.4. The next day, sections were incubated for 60 min in biotinylated goat anti-rabbit IgG (Vector Laboratories, Burlingame, CA, USA) 1:200. The antigen–antibody reaction was revealed by 60 min incubation with the biotin–avidin system (Vectastain ABC Kit Elite, Vector Laboratories, Burlingame, CA, USA). The peroxidase activity was visualized with a solution containing 0.400 mg/mL of 3,3′-diamino-benzidine (DAB, Sigma–Aldrich, Milano, Italy) and 0.004% hydrogen peroxide in 0.05 M Tris–HCl buffer, pH 7.6. Sections were mounted on chromallum-coated slides, air-dried, cleared in xylene, and cover slipped with Entellan (Merck, Milano, Italy).

The production and characterization of NPY polyclonal antibody has been previously reported [79,80] and it has been employed to detect the NPY system in a wide range of species [40].

The POMC antibody from Phoenix Pharmaceuticals recognizes a sequence corresponding to N terminal amino acids 27–52 of Pig POMC precursor and has often been used in mouse and rat studies [31,42,81].

We performed the following additional controls in our material: (a) the primary antibody was omitted or replaced with an equivalent concentration of normal serum (negative controls) and (b) the secondary antibody was omitted. In these conditions, cells and fibers were completely unstained.

### 4.3. Quantitative Analysis

All sections were acquired with a NIKON Digital Sight DS-Fi1 video camera connected to a NIKON Eclipse 80i microscope (Nikon Italia S.p.S., Firenze, Italy). The staining density of NPY- and POMC-immunoreactive (ir)-containing structures was measured in selected nuclei with the freeware ImageJ (version 1.49b, Wayne Rasband, NIH, Bethesda, MD, USA) by calculating in binary transformations of the images (threshold function) the fractional area (percentages of pixels) covered by immunoreactive structures in predetermined fields (area of interest, ROI). Due to differences in the immunostaining, according to our previous reports [50,63], the range of the threshold was individually adjusted for each section.

For quantification of NPY and POMC systems we selected four hypothalamic nuclei involved in controlling food intake—ARC, DMH, PVN, and ventromedial hypothalamic nucleus (VMH). For each nucleus, we measured the density of immunoreactive structures on three consecutive sections identified by the Mouse Brain Atlas (ARC, VMH, DMH: bregma −1.46mm, −1.58mm, −1.70mm; PVN: bregma −0.70mm, −0.82mm, −0.94mm [82,83].

The ROI selected for each nucleus was a box of fixed size and shape, selected to cover immunoreactive material only within the boundaries of each nucleus (about 140,000 μm^2^ for VMH and DMH, 110,000 μm^2^ for ARC, and 200,000 μm^2^ for PVN). Due to the extreme paucity of immunoreactive structures, it was not possible to measure POMC-immunoreactivity in the VMH.

### 4.4. Statistical Analysis

Collected data were analyzed with the program SPSS 24.0 (SPSS Inc., Chicago, IL, USA); the *p* values and the significance threshold were set at *p* ≤ 0.05. Data collected for the body weight were analyzed by one-way ANOVA followed by post-hoc analysis with a Fisher LSD test. Data collected for the immunohistochemistry were analyzed by repeated-measure one-way ANOVA. When the analysis did not show significant differences between different levels of the same nucleus, we calculated a mean value for each nucleus that was used to assess variations due to the treatment. When statistically significant, the ANOVA analysis was followed by a Fisher LSD test.

## Figures and Tables

**Table 1:** Summary of statistical analysis of body weight data. The values (in grams) are indicated as mean ± standard error of the mean (SEM). Bold numbers and asterisks indicate significant differences among the differently treated groups: * *p* < 0.05, different from control (*p* < 0.05, Fisher’s test).

Groups	Body Weight (g) Mean +/− SEM	*p* Value
CRL	31.2 ± 2.92	
TBT 0.5	31 ± 0.89	0.912
TBT 500	31.2 ± 0.80	1.000
DES 0.05	29.4 ± 1.21	0.323
DES 50	26.6 ± 0.93	0.015 *
BPA 5	28.6 ± 0.75	0.156
BPA 500	29.6 ± 0.93	0.379
E2 5	26.75 ± 0.48	0.025 *
E2 50	27.17 ± 0.65	0.025 *

## Data Availability

All the data are available from the authors upon reasonable request. The data presented in this study are available in this supplementary.

## Supplementary Materials

The following are available online at https://www.mdpi.com/article/10.3390/metabo11060368/s1, Figure S1: NPY immunoreactivity. Immunohistochemical comparison of NPY immunoreactivity among control animals (CRL) and the different treated groups (in all case it was shown to be the lowest dose used) in the dorsomedial (DMH), ventromedial (VMH), arcuate (ARC), and paraventricular (PVN) nuclei. Estradiol, E2; tributyltin, TBT; diethylstilbestrol, DES; bisphenol A, BPA. Scale bar = 100 μm, Figure S2: POMC immunoreactivity. Immunohistochemical comparison of POMC immunoreactivity among control animals (CRL) and the different treated groups (in all case it was shown to be the lowest dose used) in the dorsomedial (DMH), arcuate (ARC), and paraventricular (PVN) nuclei. Estradiol, E2; tributyltin, TBT; diethylstilbestrol, DES; bisphenol A, BPA. Scale bar = 100 μm, Figure S3: Regional analysis of POMC immunoreactivity in the PVN. To further confirm the absence of variations in the POMC expression within the PVN, we measured the immunoreactivity, according to our previous studies [54], by dividing the PVN into four quadrants: dorsomedial (DM), dorsolateral (DL), ventromedial (VM), and ventrolateral (VL). The results of this analysis reported no significant differences for all the analyzed subregions and are summarized in the histograms (B−E). Scale bar = 100 µm, Table S1. Composition of the soy-free diet (SFSD), Table S2. Summary of quantitative analysis of the fractional area. Fractional area data in the different nuclei and in the different groups analyzed in this study. The values reported are the mean and standard error of the mean (SEM). Bold numbers and asterisks indicate significant differences (Fisher’s test) among the differently treated groups: * *p* < 0.05, ** *p* < 0.01, *** *p* < 0.001 different from control.
