# Supplementary material for: Hypothalamic Expression of Neuropeptide Y (NPY) and Pro-OpioMelanoCortin (POMC) in Adult Male Mice Is Affected by Chronic Exposure to Endocrine Disruptors
Source: Metabolites. 2021 Jun 9;11(6):368. doi: 10.3390/metabo11060368 (PMC8228876; doi:10.3390/metabo11060368)
Supplement: Supplementary file 1 [file metabolites-11-00368-s001.zip › supplementary/Table 1S.pdf]

Table 1S Composition of the soy-free diet (SFSD). The diet was purchased from *Laboratorio Dottori Piccioni* (Gessate, Italy).

	Soy-free standard certified diet (SFSD) n. 48 Composition %
Moisture	12
Crude protein	22
Fat	5.4
Fiber	3.6
Ash	7.2
Cellulose	-
	Ingredients
Ground grains	+
Soybeans	-
Meat meal	+
Fish meal	+
Whey	+
Milk serum powder	+
Brewer's yeast	+
Alfa Alfa	-
Maize starch	+
Corn oil	+
Sucrose	+
Calcium carbonate	+
Chrome potassium sulphate	+
Dicalcium phosphate	+
Potassium iodate	-
Potassium sulphate	-
Sodium chloride	+
D,L-methionine	-
Casein	-
	Supplements/Kg
VITAMIN A (UI)	12000
VITAMINA D3 (UI)	1000
VITAMIN K (mg)	1
VITAMIN E (mg)	40
VITAMIN B1 (mg)	8
VITAMIN B2 (mg)	10
VITAMIN B6 (mg)	10
VITAMIN PP (mg)	40
VITAMIN B12 (mg)	0.02
Pantothenic acid (mg)	15
Folic acid (mg)	2
Choline (mg)	1000
Biotin (mg)	0.1
Cobalt (mg)	0.25
Iodine (mg)	0.8
Manganese carbonate (mg)	55
Iron citrate (mg)	100
Copper carbonate (mg)	8
Potassium citrate monohydrate (mg)	-
Sodium selenite (mg)	-
Zinc carbonate (mg)	50
