# Supplementary material for: Hypothalamic Expression of Neuropeptide Y (NPY) and Pro-OpioMelanoCortin (POMC) in Adult Male Mice Is Affected by Chronic Exposure to Endocrine Disruptors
Source: Metabolites. 2021 Jun 9;11(6):368. doi: 10.3390/metabo11060368 (PMC8228876; doi:10.3390/metabo11060368)
Supplement: Supplementary file 1 [file metabolites-11-00368-s001.zip › supplementary/Table2S.pdf]

	TREATMENT								
**nucleus**	**CRL**	**TBT 0.5**	**TBT 500**	**DES 0.05**	**DES 50**	**BPA 5**	**BPA 500**	**E2 5**	**E2 50**
**NPY**									
**DMH**	23.95 ± 1.33	**19.80 ± 2.03***	**18.30 ± 1.54****	22.16 ± 1.10	**18.26 ± 1.55****	**17.04 ± 0.73*****	**16.53 ± 1.21*****	**17.44 ± 0.09*****	**17.87 ± 0.98****
**VMH**	15.85 ± 0.32	**9.55 ± 1.03****	14.12 ± 1.19	**10.37 ± 0.73****	**9.58 ± 1.43****	**9.24 ± 1.06****	**11.16 ± 1.33***	**9.93 ± 0.59****	**10.27 ± 0.98****
**ARC**	34.74 ± 2.28	29.61 ± 3.50	**28.50 ± 0.96***	**27.03 ± 1.23***	**21.07 ± 2.43*****	**23.99 ± 1.84****	30.58 ± 2.10	**27.16 ± 2.72***	**23.36 ± 1.62*****
**PVN**	37.46 ± 1.36	**32.05 ± 1.13****	**28.56 ± 0.79*****	**27.73 ± 1.30*****	**27.29 ± 1.48*****	**25.93 ± 1.12*****	**26.42 ± 0.54*****	**26.75 ± 0.86*****	**30.27 ± 1.22*****
**POMC**									
**DMH**	5.63 ± 0.12	4.99 ± 0.17	**4.57 ± 0.19****	**4.45 ± 0.26*****	**4.29 ± 0.09*****	**3.54 ± 0.10*****	**3.24 ± 0.07**	**3.61 ± 0.20*****	**4.09 ± 0.25*****
**ARC**	4.55 ± 0.26	3.92 ± 0.28	3.65 ± 0.26	3.89 ± 0.34	**3.00 ± 0.31*****	**3.23 ± 0.41****	**2.94 ± 0.24*****	**3.01 ± 0.27*****	4.08 ± 0.28***
**PVN**	16.16 ± 1.71	17.07 ± 0.87	16.68 ± 1.18	15.40 ± 1.08	16.95 ± 0.69	14.41 ± 1.15	14.95 ± 0.80	13.52 ± 0.43	15.92 ± 1.55
