# Supplementary figures and images for: Hypothalamic Expression of Neuropeptide Y (NPY) and Pro-OpioMelanoCortin (POMC) in Adult Male Mice Is Affected by Chronic Exposure to Endocrine Disruptors
Source: Metabolites. 2021 Jun 9;11(6):368. doi: 10.3390/metabo11060368 (PMC8228876; doi:10.3390/metabo11060368)

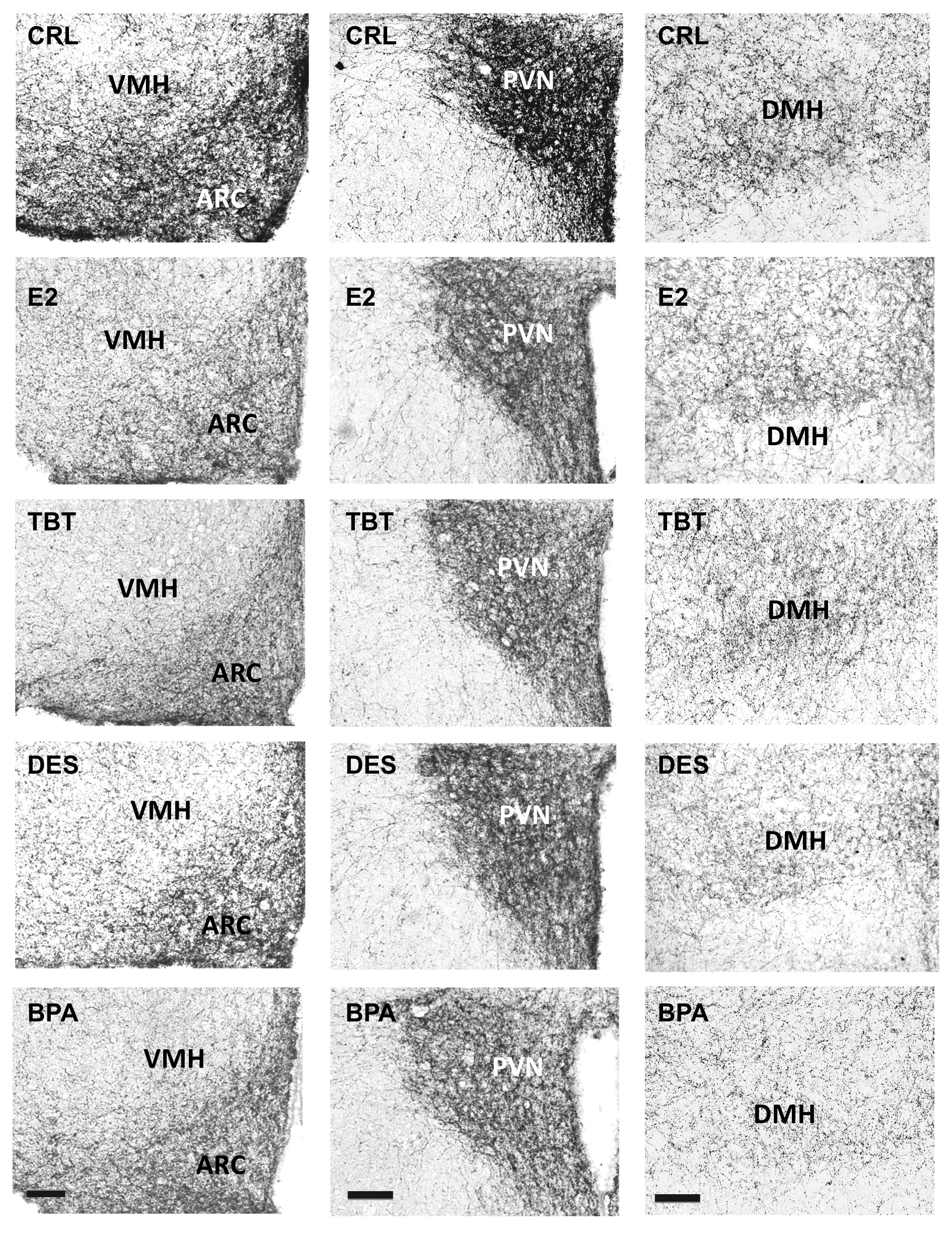

Supplement: Supplementary file 1 [file metabolites-11-00368-s001.zip › supplementary/Figure 1S.tiff]

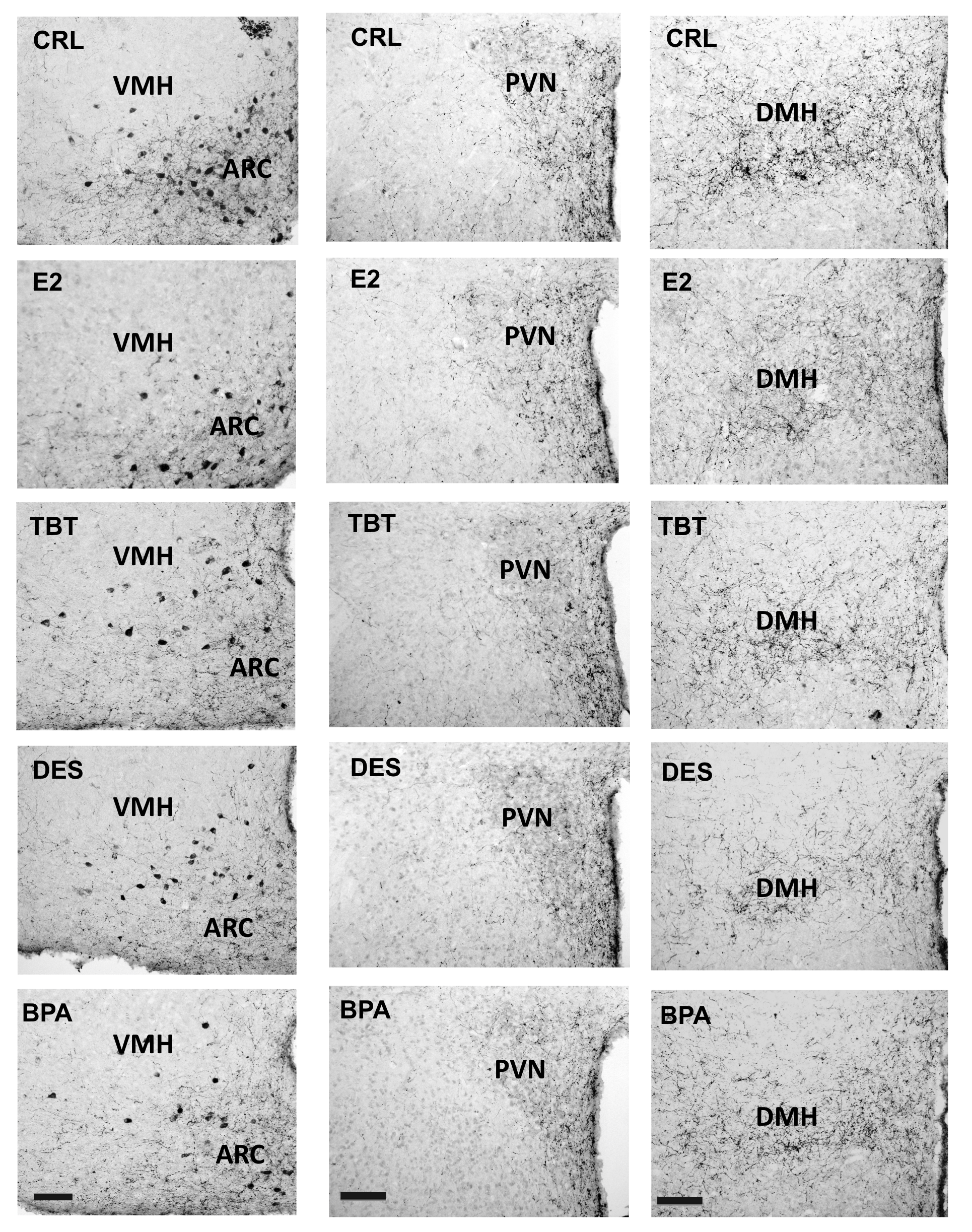

Supplement: Supplementary file 1 [file metabolites-11-00368-s001.zip › supplementary/Figure 2S.tiff]

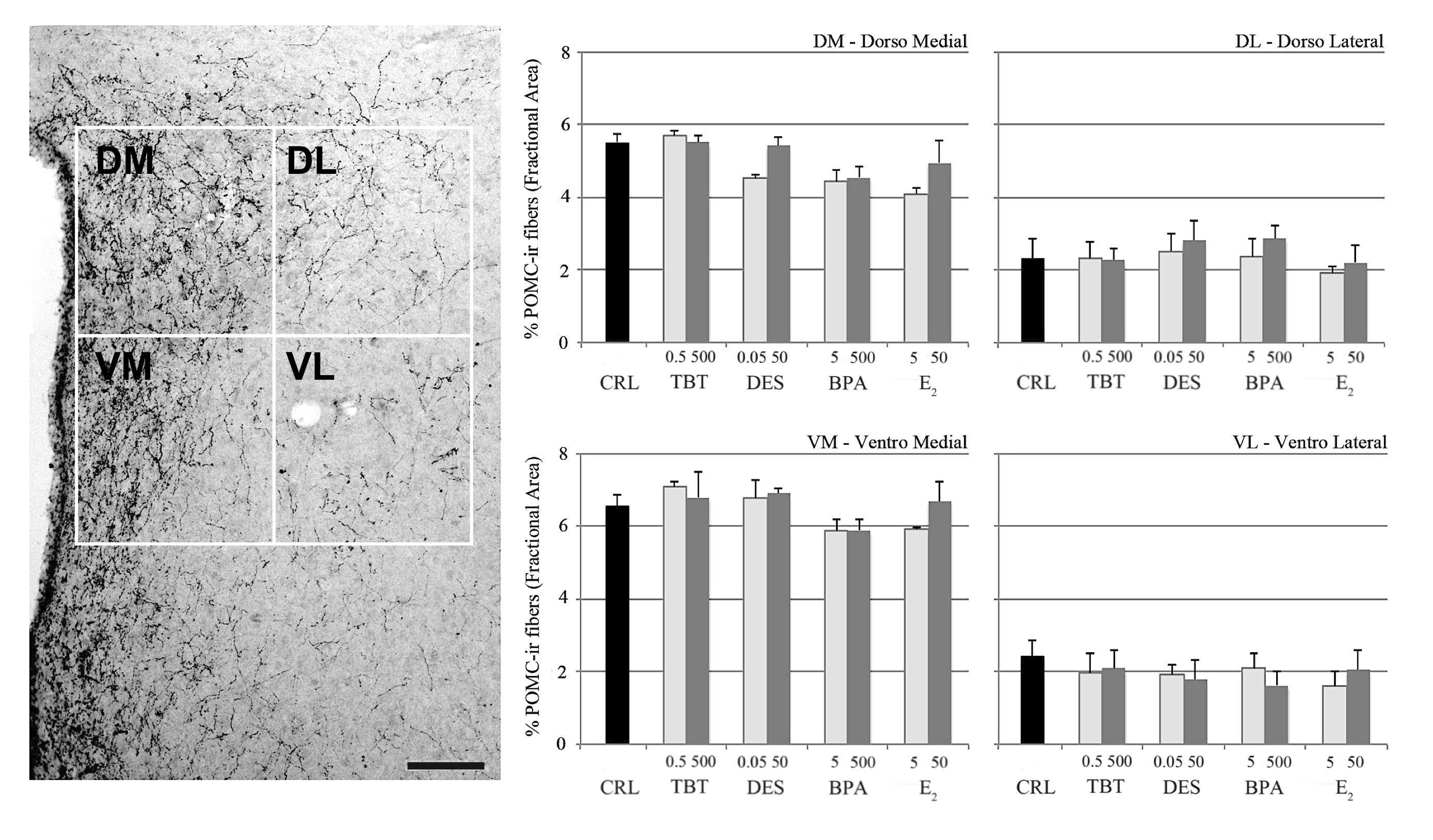

Supplement: Supplementary file 1 [file metabolites-11-00368-s001.zip › supplementary/Figure 3s.tiff]
